# The mitochondrial Ca^2+^ uniporter channel synergizes with fluid shear stress to induce mitochondrial Ca^2+^ oscillations

**DOI:** 10.1038/s41598-022-25583-7

**Published:** 2022-12-07

**Authors:** Akshar Patel, Matthew Simkulet, Soumya Maity, Manigandan Venkatesan, Anastasios Matzavinos, Muniswamy Madesh, B. Rita Alevriadou

**Affiliations:** 1 Vascular Mechanobiology Laboratory, Department of Biomedical Engineering, and Center for Cell, Gene, and Tissue Engineering, University at Buffalo – The State University of New York, Buffalo, NY 14260 USA; 2 Center for Mitochondrial Medicine, Department of Medicine, University of Texas Health San Antonio, San Antonio, TX 78229 USA; 3 Institute for Mathematical and Computational Engineering, Pontifical Catholic University of Chile, Santiago, Chile

**Keywords:** Calcium signalling, Ion channel signalling, Cellular imaging, Mitochondria, Biomedical engineering, Ca2+ imaging, Wide-field fluorescence microscopy, Applied mathematics, Atherosclerosis, Ion transport

## Abstract

The mitochondrial calcium (Ca^2+^) uniporter (MCU) channel is responsible for mitochondrial Ca^2+^ influx. Its expression was found to be upregulated in endothelial cells (ECs) under cardiovascular disease conditions. Since the role of MCU in regulating cytosolic Ca^2+^ homeostasis in ECs exposed to shear stress (SS) is unknown, we studied mitochondrial Ca^2+^ dynamics (that is known to decode cytosolic Ca^2+^ signaling) in sheared ECs. To understand cause-and-effect, we ectopically expressed MCU in ECs. A higher percentage of MCU-transduced ECs exhibited mitochondrial Ca^2+^ transients/oscillations, and at higher frequency, under SS compared to sheared control ECs. Transients/oscillations correlated with mitochondrial reactive oxygen species (mROS) flashes and mitochondrial membrane potential (ΔΨ_m_) flickers, and depended on activation of the mechanosensitive Piezo1 channel and the endothelial nitric oxide synthase (eNOS). A positive feedback loop composed of mitochondrial Ca^2+^ uptake/mROS flashes/ΔΨ_m_ flickers and endoplasmic reticulum Ca^2+^ release, in association with Piezo1 and eNOS, provided insights into the mechanism by which SS, under conditions of high MCU activity, may shape vascular EC energetics and function.

## Introduction

Calcium ion (Ca^2+^) influx into the mitochondria is driven by a large negative membrane potential (ΔΨ_m_) across the inner mitochondrial membrane (IMM) and is mediated by a low-affinity, high-capacity, Ca^2+^-selective ion channel complex called the Mitochondrial Ca^2+^ Uniporter (MCU). The MCU is a heteromeric complex that includes the Ca^2+^-conducting core protein, also called MCU, and the regulatory proteins MCU dominant-negative β-subunit (MCUb), essential MCU regulator (EMRE), the mitochondrial Ca^2+^ (_m_Ca^2+^) uptake (MICU) family (MICU1-3), MCU regulator 1 (MCUR1), and solute carrier 25A23 (SLC25A23)^1–6^. Due to the formation of mitochondria-associated membranes (MAMs), a type of quasi-synaptic junctions between the endoplasmic reticulum (ER; sarcoplasmic reticulum, SR, in the case of excitable cells) and neighboring mitochondria, Ca^2+^ concentration in the MAM region can reach sufficiently high levels to activate the MCU complex and lead to efficient _m_Ca^2+^ uptake^7–9^. Besides being regulated by the Ca^2+^ concentration in MAMs and, in essence, by the cytosolic Ca^2+^ (_c_Ca^2+^) concentration ([Ca^2+^]_c_), the complex activity is also regulated by the levels of reactive oxygen species (ROS) in the mitochondrial matrix (mROS), via a posttranscriptional modification of the MCU protein tail that faces the matrix, and by a continuously increasing number of transcriptional, posttranscriptional, and posttranslational modifications of its subunits^6,10^. In return, MCU complex activity regulates the mitochondrial free Ca^2+^ concentration ([Ca^2+^]_m_), and, hence, plays a key role in mitochondrial respiration, ATP production, mitophagy/autophagy, and the mitochondrial pathway of apoptosis^11–13^. Furthermore, the _m_Ca^2+^ fluxes (uptake via the MCU, and extrusion via the mitochondrial Na^+^/Ca^2+^ exchanger, mNCX) shape the spatiotemporal profiles of [Ca^2+^]_c_ and regulate all Ca^2+^-dependent cell functions, including activation of kinases/phosphatases, gene transcription, and cell survival^6,14–17^.

MCU gene/protein expression (and, hence, the MCU complex activity) in cultured human umbilical vein endothelial cells (HUVECs) was found to be necessary in maintaining the [Ca^2+^]_c_ oscillations during endothelial cell (EC) exposure to arterial-level steady laminar shear stress (SS), an in vitro flow condition that mimics the in vivo hemodynamic environment in straight segments of arteries and arterioles and contributes to the anti-inflammatory and atheroresistant EC phenotype in those regions of the vasculature^18–23^. Recent studies showed the EC MCU gene/protein to be upregulated under pathological conditions associated with cardiovascular disease (CVD). Specifically, in apolipoprotein E-knockout (apoE^−/−^) mice fed a high fat diet and in cultured HUVECs treated with hydrogen peroxide (H_2_O_2_), EC MCU expression was upregulated leading to _m_Ca^2+^ overload and cell apoptosis^24^. HUVEC treatment with either homocysteine or high glucose also upregulated MCU expression/increased MCU channel activity and led to EC dysfunction and apoptosis^25,26^. These findings suggest that altered _m_Ca^2+^ influx may play a key role in EC dysfunction, the earliest and most critical step in CVD development^27,28^.

Based on the above, we designed experiments to monitor the (currently unknown) [Ca^2+^]_m_ temporal profiles in SS-exposed HUVECs and test the hypothesis that the SS-induced _m_Ca^2+^ response may be altered following EC MCU overexpression (OX). Furthermore, we aimed to delineate the underlying molecular mechanisms responsible for any alterations in the SS-induced EC _m_Ca^2+^ response due to MCU OX. Using the mitochondria-targeted genetically-encoded Ca^2+^ indicator (GECI) mito-GCaMP6(m) and mathematical analysis of fluorescence imaging data, we discovered that MCU-transduced ECs when exposed to SS exhibit [Ca^2+^]_m_ oscillations at a frequency of ~ 10 mHz. There were significantly fewer [Ca^2+^]_m_ transients in untransduced (or βGal-transduced) ECs exposed to SS for the same time of exposure, and barely any [Ca^2+^]_m_ transients in either MCU-transduced or untransduced (or βGal-transduced) ECs kept under static conditions. By employing appropriate pharmacological agents and based on published work by others, we proposed a molecular pathway where MCU OX synergizes with SS to cause mitochondrial ROS (mROS) generation, transient ΔΨ_m_ depolarization and mitochondrial permeability transition pore (mPTP) opening, followed by mROS release into the MAMs and sensitization of the ER inositol 1,4,5-trisphosphate receptor (IP_3_R). The released Ca^2+^ from the ER, via MCU-mediated _m_Ca^2+^ uptake, generates each subsequent [Ca^2+^]_m_ oscillation. The plasma membrane mechanosensitive Ca^2+^ channel Piezo1 and the SS-produced nitric oxide (NO) were involved in maintenance of the above _m_Ca^2+^ signaling pathway. In summary, our study showed that increased MCU activity disrupts the normal Ca^2+^ signaling in response to arterial-level SS by causing [Ca^2+^]_m_ oscillations, which may predispose the vascular endothelium to mitochondrial oxidative stress, inflammation and apoptosis, and contribute to CVD initiation.

## Results

### MCU abundance determines the _m_Ca^2+^ dynamics in ECs under SS

**Figure 1:**
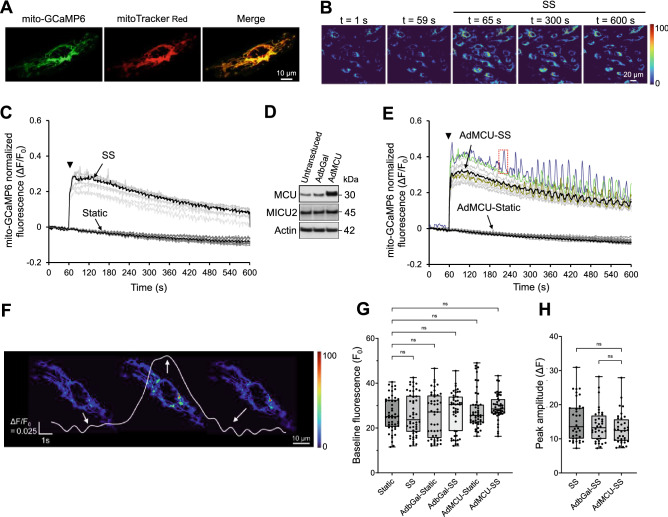
MCU OX causes [Ca^2+^]_m_ transients/oscillations in sheared HUVECs. (**A**) A characteristic fluorescence image of mito-GCaMP6 shows overlap with the mitochondrial network, identified by mitoTracker Red, in an EC. (**B**) Characteristic mito-GCaMP6 microscope images of an EC monolayer at different times, while exposed to 1 min static followed by 9 min of SS. (**C**) Characteristic temporal profiles of normalized mito-GCaMP6 fluorescence for each EC in a microscope field of view, while exposed to either static incubation for 10 min (Static) or 1 min static incubation followed by 9 min of SS (SS). (**D**) A characteristic Western blot of lysates from untransduced, Ad.βGal- or Ad.MCU-transduced (all static) ECs against MCU, MICU2, and β-actin. Original/uncropped blots are presented in Supplementary Fig. 1 (15 µg total protein per lane). (**E**) Same as in (**C**), but for Ad.MCU-transduced ECs. (**F**) Magnification of a [Ca^2+^]_m_ transient (dotted box in **E**). (**G**) Box plots of baseline fluorescence (F_0_) in arbitrary units and (**H**) of peak amplitude (ΔF) were plotted and statistically analyzed for 60 ECs (cells were from n = 4 independent experiments) in each condition tested; ns, P > 0.5.

Genetically-expressed mito-GCaMP6^29,30^ properly overlapped with mitoTracker Red in HUVECs (Fig. 1A; digital magnification from a 63 × fluorescence microscope image). When monolayers were subjected to 1 min static incubation followed by 9 min of exposure to arterial-level SS, mito-GCaMP6 fluorescence in each EC in the field of view remained unchanged during the 1 min static (Fig. 1B; 1 and 59 s), rose within 1 s after the onset of flow, maintained the high level of fluorescence for at least the first min of SS (Fig. 1B; 65 s) and slowly declined with time during SS exposure (Fig. 1B; 300 and 600 s). Temporal profiles of normalized, average for each ROI (each EC is treated as an ROI), mito-GCaMP6 fluorescence intensity (ΔF/F_0_ vs. time) showed that all ECs in the monolayers responded with a sharp increase in mito-GCaMP6 fluorescence (an index of [Ca^2+^]_m_) upon the onset of flow (Fig. 1C; Supplementary Video 1), whereas ECs that were maintained static throughout the 10 min period maintained their fluorescence with a slow decline (Fig. 1C). The thick black lines represent the average normalized fluorescence for all cells at each time point. When ECs were transduced with an adenoviral vector to OX MCU (Fig. 1D; Supplementary Fig. 1), as before^31^, and were exposed to SS (Ad.MCU-SS), they exhibited the same temporal profile of ΔF/F_0_ as the sheared untransduced ECs, which comprised of a steady ΔF/F_0_ during the 1 min static followed by a sharp rise upon the onset of SS (Fig. 1E). However, several MCU OX cells also exhibited pronounced [Ca^2+^]_m_ transient responses with an oscillatory pattern during the 9 min of SS (Fig. 1E; Supplementary Video 2). These responses were absent in ECs transduced to OX MCU and kept static for 10 min (Ad.MCU-Static; Fig. 1E). Ad.βGal-transduced ECs responded to SS (not shown) exactly as the sheared untransduced ECs (Fig. 1C). A typical transient from the temporal profile of an Ad.MCU-SS cell (dotted box; Fig. 1E) had an amplitude of ~ 0.15 ΔF/F_0_ and a duration of ~ 5 s (Fig. 1F). No significant differences in baseline fluorescence (F_0_, in arbitrary units) were found between (untransduced) Static and each of the other conditions (SS, Ad.βGal-Static, Ad.βGal-SS, Ad.MCU-Static, Ad.MCU-SS) following analysis of 60 ECs per condition, as well as among all conditions tested (Fig. 1G). Furthermore, MCU OX did not cause any significant differences in peak amplitude (ΔF; the increase in F that occurs upon the onset of SS) compared to either (untransduced) SS or Ad.βGal-SS (Fig. 1H).

### Quantitation of [Ca^2+^]_m_ transients in a cell using mathematical analysis

**Figure 2:**
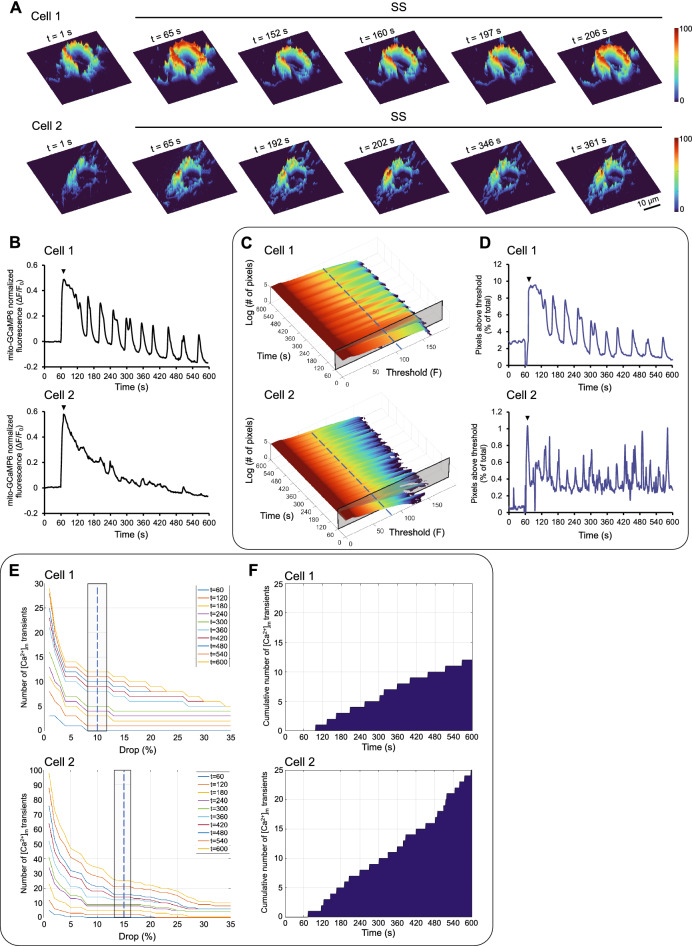
Mathematical analysis to identify [Ca^2+^]_m_ transients. (**A**) Pseudo-3D fluorescent images of two extreme cases: Cell 1 exhibited [Ca^2+^]_m_ transients in ~ 10% of its area, whereas cell 2 in ~ 1% of its area. (**B**) The ΔF/F_0_ temporal profile for cell 1 identified the transients; it did not identify them in the case of cell 2. (**C**) 3D topography surfaces of log_10_(number of pixels) vs. threshold intensity vs. time were constructed using MATLAB to assist in selecting the best threshold value for each cell. (**D**) Using the chosen threshold value, the number of pixels above the threshold intensity (as a percentage of total cell area) was plotted vs. time for each cell. (**E**) The numbers of [Ca^2+^]_m_ transients were plotted as a function of different drop percentage values and times for each cell, and the drop value that yielded the most persistent transients was chosen. (**F**) Using the chosen threshold and drop values, the cumulative number of transients was plotted vs. time (1 min static followed by 9 min SS) for each cell.

Whereas a limited number of MCU OX cells, such as cell 1 in Fig. 2A, had a large region of their mitochondrial network undergoing synchronized [Ca^2+^]_m_ oscillations during SS (pseudo-3D images show the initial peak in mito-GCaMP6 fluorescence after the onset of SS and two additional peaks at later time points, 160 and 206 s), there were many cells that fell in the opposite category. These cells, such as cell 2 in Fig. 2A, had only small regions of their mitochondrial network exhibit [Ca^2+^]_m_ oscillations (pseudo-3D images show the initial peak and two additional peaks at later time points, 202 and 361 s). As a result, the temporal profile of normalized, averaged over the cell area (cell’s mitochondrial network) mito-GCaMP6 fluorescence intensity (ΔF/F_0_) identified the [Ca^2+^]_m_ oscillations in cell 1, but it was unable to identify them in cell 2 (Fig. 2B). By employing the MATLAB function *nnz*, the total number of pixels above a threshold intensity was measured for a range of thresholds throughout the 10 min period (1 min static and 9 min SS) and was plotted as a 3D topography surface for each cell (Fig. 2C; the z axis shows the total number of pixels above the threshold in a logarithmic scale). Thresholds were then selected by visual inspection of the 3D topography surface to maximize the number of discrete [Ca^2+^]_m_ transients (a threshold of 110 was selected for cell 1 and a lower threshold, 80, was selected for cell 2; Fig. 2C). Plotting the temporal profile of the total number of pixels above the selected threshold (normalized to the total pixels of the cell area) allowed for identification of the same, as before, transients in cell 1 (compare cell 1 in Fig. 2D vs. 2B), but it also allowed for identification of transients in cell 2 (compare cell 2 in Fig. 2D vs. 2B). By employing the MATLAB function *findpeaks* with the parameter *MinPeakProminence*, [Ca^2+^]_m_ transients were counted and plotted as a function of drop vs. time every 60 s for each cell (Fig. 2E), where a drop was defined as the percentage of maximum normalized fluorescence intensity (the maximum occurs right after the onset of SS) by which the relative maxima must be greater than the preceding relative minima. The drop value was chosen as the one that produced the most persistent [Ca^2+^]_m_ transients (the selected drop value was 10 for cell 1 and 15 for cell 2; Fig. 2E). Using the chosen threshold and drop values, the total number of [Ca^2+^]_m_ transients during the 9 min SS was determined for cell 1 and 2 (12 and 25 total [Ca^2+^]_m_ transients, respectively; Fig. 2F). These numbers were verified by detailed visual inspection of mito-GCaMP6 videos, confirming the validity of our mathematical analysis. Our in-house developed MATLAB code (Supplementary Code) was employed throughout this study to quantify the [Ca^2+^]_m_ transients for each cell in each condition tested.

### MCU OX causes mROS and ΔΨ_m_ transients in ECs under SS

**Figure 3:**
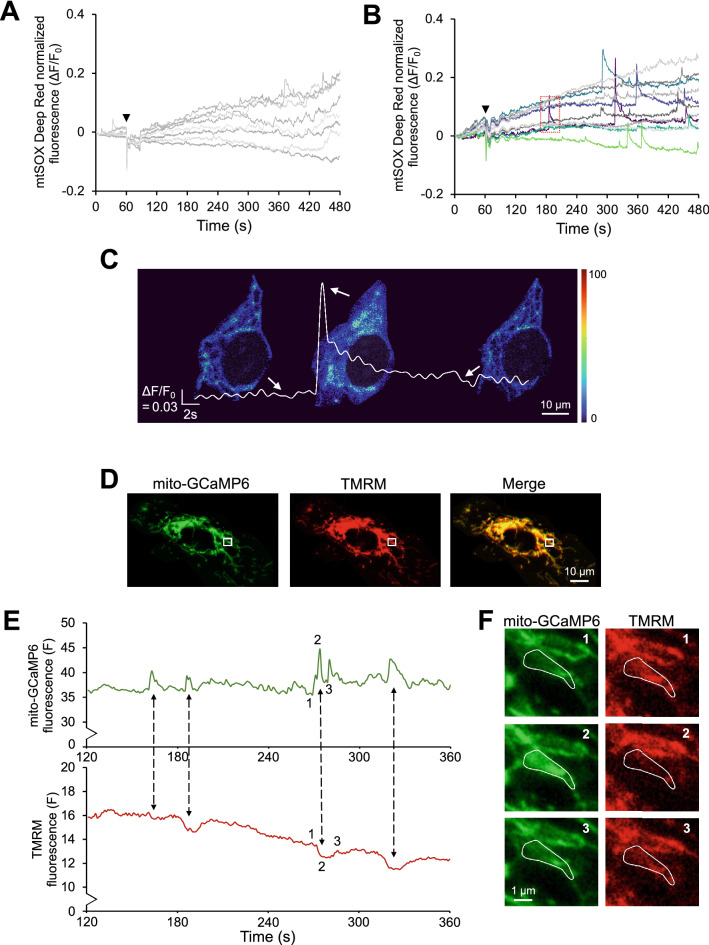
MCU OX causes mROS flashes and ΔΨ_m_ flickers in sheared ECs. (**A**) Characteristic temporal profiles of normalized mtSOX Deep Red fluorescence for each EC in a microscope image, where the monolayer was exposed to 1 min static followed by 9 min SS. (**B**) Same as in (**A**), but for Ad.MCU-transduced ECs. (**C**) Magnification of a ΔΨ_m_ transient (shown by a dotted box in **B**). (**D**) Characteristic TMRM fluorescence shows overlap with mito-GCaMP6 in an unstimulated EC. (**E**) F (in arbitrary units) averaged over an area of a cell (white box in **D**) for both mito-GCaMP6 and TMRM vs. time. (**F**) Magnification of the cell area (white box in **D**) shows opposing behavior between mito-GCaMP6 and TMRM at a single-mitochondrion level at time points 1–3 (time points shown in **E**).

To examine the potential link between [Ca^2+^]_m_ transients and mROS flashes, EC monolayers were loaded with the mitochondrial O_2_^-.^ fluorescent indicator mtSOX Deep Red. Although the mtSOX Deep Red excitation/emission spectrum is compatible for double-staining with mito-GCaMP6, when mtSOX Deep Red was loaded in ECs transduced with Ad.mito-GCaMP6, the mitochondrial network underwent morphological changes, which prevented measurement of mito-GCaMP6 and mtSOX Deep Red fluorescence from the same cells. When untransduced (or Ad.βGal-transduced) ECs loaded with mtSOX Deep Red were exposed to SS, they showed an increasing trend in fluorescence with a few random transient peaks during SS (Fig. 3A). In contrast, when Ad.MCU-transduced ECs loaded with mtSOX Deep Red were exposed to SS, they exhibited, besides the rising trend, some large-magnitude transients (mROS flashes) that were absent during the 1 min static (Fig. 3B). A typical mtSOX Deep Red transient of an MCU-transduced cell under SS (dotted box; Fig. 3B) had an amplitude of ~ 0.2 ΔF/F_0_ and a duration of ~ 6 s (Fig. 3C), close to the measurements of a typical mito-GCaMP6 transient (Fig. 1F). Since work by others showed that mROS flashes are associated with ΔΨ_m_ flickers in smooth muscle cells, cardiomyocytes, and cancer cells^32,33^, we labeled MCU- and mito-GCaMP6-transduced ECs with the ΔΨ_m_ indicator tetramethylrhodamine methyl ester (TMRM) that accumulates only in active mitochondria^34^, and monitored both the mito-GCaMP6 and TMRM fluorescence in the same cells over time under SS. TMRM staining was shown to properly overlap with mito-GCaMP6 staining in static ECs at baseline in the absence of any mechanical or chemical treatment (Fig. 3D). Temporal profiles in fluorescence (F, in arbitrary units) for each of the fluorophores averaged over a region within a cell (i.e., white box; Fig. 3D) showed that each transient increase in mito-GCaMP6 fluorescence occurred simultaneously with a transient decline in TMRM fluorescence (Fig. 3E), suggesting that the [Ca^2+^]_m_ transients/oscillations in sheared MCU OX ECs are associated with ΔΨ_m_ flickers. When the selected region was digitally magnified, fluorescent images of mito-GCaMP6 and TMRM from a single mitochondrion at three different time frames during SS (time points were indicated in Fig. 3E) showed an opposite behavior between the two indicators at the single-mitochondrion level (Fig. 3F).

### Piezo1, mROS, and NO are responsible for the SS-induced _m_Ca^2+^ response

**Figure 4:**
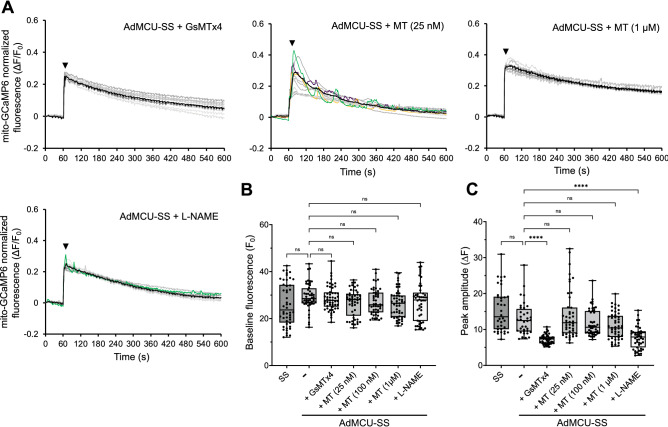
Piezo1-mediated Ca^2+^ influx, mROS and NO signaling determine the _m_Ca^2+^ response in sheared MCU OX ECs. (**A**) Characteristic temporal profiles of normalized mito-GCaMP6 fluorescence of MCU OX ECs in a microscope field of view that were exposed to 1 min static followed by 9 min of SS in the presence of either GsMTx4 (5 µM), MT (either 25 nM or 1 µM), or L-NAME (500 µM). The temporal profile of MCU OX ECs in a microscope field of view exposed to the same protocol, but in the absence of any compound, was shown in Fig. 1E. (**B**) None of the tested compounds, GsMTx4, MT (25 nM, 100 nM or 1 µM), or L-NAME, had a significant effect on baseline fluorescence F_0_ of sheared MCU-transduced ECs. F_0_ of sheared untransduced ECs is also shown. A total of 60 cells (from n = 4 independent experiments) per condition were analyzed. (**C**) GsMTx4 and L-NAME, but not MT, significantly decreased the peak magnitude ΔF (right after the onset of SS) of sheared MCU-transduced ECs. ΔF of sheared untransduced ECs is also shown. A total of 60 cells (from n = 4 independent experiments) per condition were analyzed.

Since Piezo1 is known to mediate the initial elevation in [Ca^2+^]_c_ in ECs exposed to SS^35,36^, we examined whether Piezo1-mediated Ca^2+^ influx was necessary for the initial rise in [Ca^2+^]_m_ and the subsequent [Ca^2+^]_m_ oscillations by both preincubating and exposing MCU-transduced ECs to SS in the presence of the Piezo1 inhibitor Grammostola spatulata Mechanotoxin 4 (GsMTx4, D-isoform) at a final concentration of 5 µM^37,38^. The D-isoform of GsMTx4 at 3 µM was previously shown to inhibit whole cell Piezo1 currents by 70 ± 6%^37^. A characteristic ΔF/F_0_ temporal profile of MCU-transduced cells in the presence of GsMTx4 is shown in Fig. 4A: GsMTx4 abolished the occurrence of SS-induced [Ca^2+^]_m_ transients compared to that in MCU OX cells exposed to SS in the absence of GsMTx4 (Fig. 1E), suggesting that Piezo1-mediated Ca^2+^ influx is critical for the generation of SS-induced [Ca^2+^]_m_ oscillations. GsMTx4 did not affect the baseline fluorescence F_0_ (Fig. 4B), but it significantly inhibited the peak amplitude ΔF (Fig. 4C) compared to MCU-transduced ECs sheared in the absence of GsMTx4, suggesting that Piezo1-mediated Ca^2+^ influx is, at least in part, responsible for the initial peak in the SS-induced _m_Ca^2+^ response.

ΔΨ_m_ flickers induced under cellular stress conditions (i.e., in the presence of staurosporine or oligomycin) were previously shown to lead to local mROS flashes in the mitochondrial matrix and transient ROS emissions in the MAMs, where they sensitized the IP_3_R to release ER Ca^2+^ and increase [Ca^2+^]_m_ in a cancer cell line^33^. To examine whether mROS play a role in the initial [Ca^2+^]_m_ peak and the subsequent transients, MCU-transduced ECs were both preincubated and exposed to SS in the presence of the mitochondrial superoxide (O_2_^-.^) scavenger 2-[2,2,6,6-tetramethylpiperidin-1-oxyl-4-ylamino]-2-oxoethyl triphenylphosphonium (mitoTEMPO, abbreviated MT) at either 25 nM, 100 nM, or 1 µM, concentrations known to effectively scavenge O_2_^-.^ in activated ECs^39,40^. Characteristic temporal profiles of mito-GCaMP6 ΔF/F_0_ in MCU-transduced cells exposed to SS in the presence of the lowest (25 nM) and highest (1 µM) MT concentrations tested are shown in Fig. 4A: MT inhibited the occurrence of [Ca^2+^]_m_ transients in a dose-dependent manner compared to that in MCU-transduced cells sheared in the absence of MT (Fig. 1E), suggesting that mROS play an important role in the generation of SS-induced [Ca^2+^]_m_ transients. MT did not affect significantly either F_0_ (Fig. 4B) or ΔF (Fig. 4C) compared to F_0_ and ΔF, respectively, of MCU-transduced ECs sheared in the absence of MT.

SS-induced NO was previously shown, either on its own or via the formation of peroxynitrite (ONOO^−^) in the mitochondrial matrix, to increase the EC mROS production^41–43^. To examine the role of NO in the initial [Ca^2+^]_m_ peak and subsequent oscillations, ECs were both pretreated and sheared in the presence of the NO synthase (NOS) inhibitor L-N^G^-nitro arginine methyl ester (L-NAME) at a final concentration of 500 µM, previously shown to inhibit the SS-induced endothelial NOS (eNOS) activation/NO generation^44^. A characteristic temporal profile of mito-GCaMP6 ΔF/F_0_ in MCU-transduced cells sheared in the presence of L-NAME is shown in Fig. 4A: L-NAME inhibited the occurrence of SS-induced [Ca^2+^]_m_ transients compared to that in MCU-transduced cells sheared in the absence of L-NAME (Fig. 1E), suggesting that NO is, at least in part, responsible for the generation of SS-induced [Ca^2+^]_m_ transients. L-NAME did not affect F_0_ (Fig. 4B), but it significantly inhibited ΔF (Fig. 4C), suggesting that NO is also, at least in part, responsible for the [Ca^2+^]_m_ peak at the onset of SS.

**Figure 5:**
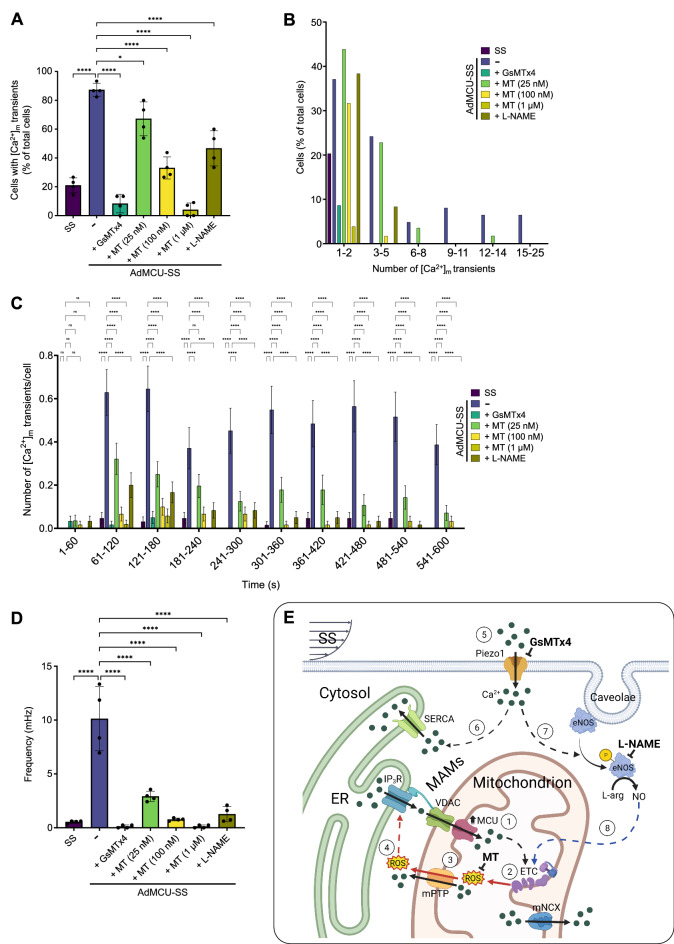
Quantitation of [Ca^2+^]_m_ transients under each condition and a proposed _m_Ca^2+^ signaling pathway in sheared MCU OX ECs. (**A**) The percentage of MCU OX cells that exhibited [Ca^2+^]_m_ transients under SS significantly decreased in the presence of either GsMTx4, MT (in a dose-dependent manner), or L-NAME (mean ± SE from n = 4 independent experiments/condition). The percentage was significantly higher in MCU OX cells (greater than 80%) compared to that in untransduced or AdβGal-transduced ECs (~ 20%). (**B**) The percentage of ECs that exhibited transients in each condition was binned over the total number of transients (excluding the initial peak that was present in every cell exposed to SS). (**C**) The total number of transients per cell was binned over 60 s intervals up to 10 min (1 min static followed by 9 min SS). Data are shown as mean ± SE for 60 cells (from n = 4 independent experiments). Only the MCU OX cells, and to a lesser extent the MCU OX cells in the presence of the lowest MT dose, exhibited persistent transients/oscillations during SS. (**D**) The average oscillation frequency (mHz) was calculated from n = 4 independent experiments per condition. ns, P > 0.5; *, P ≤ 0.05; **, P ≤ 0.01; ***, P ≤ 0.001; ****, P ≤ 0.0001. (**E**) Upregulated MCU expression leads to increased _m_Ca^2+^ influx (step 1) that increases O_2_^-.^ production by the ETC and mROS levels in the matrix (step 2). High [Ca^2+^]_m_ (and mROS) activate/open the low conductance mPTP channel (step 3). Due to redistribution of ions, mitochondria undergo a brief depolarization (ΔΨ_m_ flicker) that contributes to additional mROS generation, but also causes ROS emissions into the MAMs (step 4), where ROS sensitize the IP_3_R to release ER Ca^2+^ leading to _m_Ca^2+^ uptake via the MCU (through the voltage-dependent anion channel, VDAC) and generating the next [Ca^2+^]_m_ peak (step 1). When the low conductance mPTP opens, opening is brief, because the resultant reduction in [Ca^2+^]_m_ (and lowering of matrix pH due to entry of H^+^ ions) favors mPTP closure and, hence, steps 1 through 4 (and back to 1) take place in an oscillatory fashion. SS activates Piezo1 (step 5) and Ca^2+^ entering the cytosol maintains Ca^2+^ in the MAMs, but it may also help maintain the ER Ca^2+^ content (step 6). SS-induced Piezo1-mediated Ca^2+^ influx also activates eNOS (step 7) and the produced NO, either on its own or via generation of RNS, enhances mROS production by the ETC (step 8).

For a comprehensive quantitative analysis of [Ca^2+^]_m_ transients in the absence and presence of each of the tested compounds, we employed the mathematical analysis (as shown in Fig. 2) and first measured the percentage of total cells that exhibited transients from n = 4 independent experiments in each condition: In the presence of GsMTx4, the percentage of MCU-transduced ECs that exhibited SS-induced transients declined from greater than 80% to ~ 10%, a value slightly lower than the percentage of untransduced (or Ad.βGal-transduced) ECs that exhibited SS-induced transients (Fig. 5A), suggesting that the Piezo1-mediated Ca^2+^ influx is necessary for [Ca^2+^]_m_ transients in sheared MCU-transduced cells and that it may even contribute to the formation of the sparsely observed [Ca^2+^]_m_ transients in sheared untransduced ECs. MT dose-dependently and significantly (at each of the three concentrations tested) inhibited the percentage of ECs with [Ca^2+^]_m_ transients, confirming the role of mROS in generating SS-induced [Ca^2+^]_m_ transients in MCU OX cells and, based on the effect of the highest MT concentration, even in untransduced ECs (Fig. 5A). Last, L-NAME significantly inhibited the percentage of ECs with [Ca^2+^]_m_ oscillations, confirming the role of NO in the _m_Ca^2+^ response of sheared MCU-transduced ECs (Fig. 5A).

In continuing with the quantitative analysis of [Ca^2+^]_m_ transients in the absence and presence of each of the compounds, ECs that exhibited transients from n = 4 independent experiments per condition were binned according to their total number of transients: All the ECs with transients (~ 20% of total cells) from the untransduced (or βGal-transduced) monolayers showed 1–2 transients during the 9 min SS, excluding the initial peak at the onset of SS (Fig. 5B). In contrast, less than half of the MCU-transduced ECs with transients had 1–2 transients. The rest of them exhibited a higher number of transients (up to 25) during the 9 min of SS, with most cells having 3–5 transients (Fig. 5B). Each compound tested on MCU OX ECs was found to inhibit the percentage of ECs with higher than 1–2 transients, with the low MT concentration being the least effective (Fig. 5B).

To examine the distribution of transients over time under SS, the total number of transients per cell for all 60 cells in n = 4 independent experiments per condition was binned over 60 s intervals: It was found that the transients persisted with time under SS in the MCU-transduced ECs at a rate of ~ 0.6 transients/min (Fig. 5C). Each compound tested, except for the low MT dose, blocked the formation of transients with increasing time under SS (Fig. 5C). Last, from the total number of transients and the SS duration (9 min), a [Ca^2+^]_m_ oscillation frequency was calculated for each condition: A frequency of ~ 10 mHz was calculated for sheared MCU OX ECs, and each compound tested significantly decreased that oscillation frequency (Fig. 5D). The oscillation frequencies for sheared MCU-transduced in the presence of either GsMTx4, MT (100 nM and 1 µM), or L-NAME were not significantly different from the frequency calculated for sheared untransduced (or βGal-transduced) ECs (Fig. 5D).

## Discussion

In this study, we unveiled the _m_Ca^2+^ response in human ECs while under exposure to arterial-level SS: Using the mitochondria-targeted GECI mito-GCaMP6, we demonstrated that ECs respond to SS by an initial sharp increase in [Ca^2+^]_m_ followed by a slow decline. Importantly, in the case where the EC MCU complex activity was enhanced to mimic cardiovascular conditions, such as atherosclerosis, diabetes, or high plasma homocysteine levels, we found that SS synergizes with MCU to generate small, repetitive, transient [Ca^2+^]_m_ peaks ([Ca^2+^]_m_ oscillations) in over 80% of the cells following the initial large [Ca^2+^]_m_ peak that all ECs exhibited at the onset of SS. In comparison, SS alone (without MCU OX) caused only a few [Ca^2+^]_m_ transients in a small percentage of ECs, whereas no [Ca^2+^]_m_ transients were observed in static MCU-transduced ECs. Our findings using TMRM and mtSOX Deep Red suggested that SS synergizes with MCU to also generate ΔΨ_m_ flickers (simultaneously and at the same mitochondrial location with [Ca^2+^]_m_ transients) and mROS flashes. Our findings using GsMTx4, MT, and L-NAME demonstrated that the SS-induced oscillatory _m_Ca^2+^ response is mediated by the mechanosensitive Piezo1 channel, mROS, and eNOS activation/NO production, providing additional insights on the intracellular signaling that may lead to EC dysfunction under conditions of increased MCU channel activity.

It is noteworthy that, by employing the adenovirus-mediated MCU overexpression method, we achieved a ~ 2.5-fold MCU protein overexpression in HUVECs (Fig. 1D), which is well within the range of levels of MCU upregulation observed in tissues collected from the aortic root to the iliac artery of apoE^−/−^ mice fed a high fat diet for 12 weeks compared to mice fed a general diet (twofold)^24^, as well as compared to H_2_O_2_ (750 µM, 24 h)-treated HUVECs (2.5-fold)^24^, homocysteine (800 µM, 24 h)-treated HUVECs (twofold)^25^, and high glucose (30 mM, 72 h)-treated HUVECs (1.5-fold)^26^, in each case, compared to their respective controls.

Recent studies have explored the interrelationship among ΔΨ_m_, mROS, and [Ca^2+^]_c_ or [Ca^2+^]_m_ in different cell types under stress: Using fluorescent probes targeted to the MAM area of a cancer cell line together with super resolution microscopy, cellular stress (due to cell treatment with either the mitochondrial ATP synthase inhibitor oligomycin or the apoptosis inducer staurosporine) was shown to cause transient mPTP openings that were associated with, and/or led to, ΔΨ_m_ flickers, mROS flashes, and miniature ROS bursts in the MAMs. The latter, by oxidizing the ER-mitochondria interface, sensitized the IP_3_R to release Ca^2+^ from the ER, followed by MCU-mediated Ca^2+^ uptake and an increase in [Ca^2+^]_m_^33^. Either MCU knockout (KO) or IP_3_R KO inhibited the ΔΨ_m_ flickering suggesting a positive feedback loop^33^. Although their study did not detect [Ca^2+^]_m_ transients, studies in cardiomyocytes activated by a laser flash found correlations among ΔΨ_m_ flickering, mitochondrial O_2_^-.^ production, and [Ca^2+^]_c_ spark frequency^45^. The antioxidant N-acetyl-cysteine (NAC) was shown to block both the transient ΔΨ_m_ depolarization events^32,45^ and the [Ca^2+^]_c_ sparks^45^, collectively suggesting that mROS drive the transient mPTP openings and, by modulating the local redox environment, the [Ca^2+^]_c_ sparks. In cardiomyocytes under pressurized flow, Kim et al. showed that shear-induced [Ca^2+^]_c_ sparks were mediated by mROS through NADPH oxidase (Nox)2 activity, since they were inhibited by either MT or a Nox2 inhibitory peptide^46^. Kuznetsov et al. found a synchronicity in mROS flashes, ΔΨ_m_ flickers, and [Ca^2+^]_m_ transients in a photoactivated cancer cell line, and showed that ROS scavenging, by either Trolox or manganese O_2_^−.^ dismutase (MnSOD) OX, prevents the ΔΨ_m_ flickers^47^. Last, in HeLa cells activated by hyperosmotic stress, Hou et al. showed that both mROS and [Ca^2+^]_m_ needed to increase above a certain level to generate [Ca^2+^]_m_ transients^48^.

Our study showed that a combination of SS stimulation with increased MCU activity is required to generate [Ca^2+^]_m_ transients, which supports the notion that both mROS and [Ca^2+^]_m_ increases contribute to the oscillatory _m_Ca^2+^ response. SS alone is known to increase mROS levels in (untransduced) ECs^42,49^, and the present study showed that it also increases [Ca^2+^]_m_ (Fig. 1C). MCU OX did not change the basal [Ca^2+^]_m_, and the onset of SS caused the same [Ca^2+^]_m_ peak magnitude independently of MCU OX. However, the combination of SS and MCU OX significantly enhanced the generation of [Ca^2+^]_m_ transients compared to that in SS-exposed untransduced (or βGal-transduced) ECs. Genetic ablation of MCU exhibited lower mROS levels in primary hepatocytes^30^ tempting to speculate that MCU OX may cause an increase in mROS, and, when SS stimulation is added, the levels of mROS and [Ca^2+^]_m_ may satisfy the requirements for generation of [Ca^2+^]_m_ transients.

The inhibitory effect of MT on the [Ca^2+^]_m_ transients confirmed the central role of mROS in the positive feedback loop inside sheared MCU-transduced ECs: Increased [Ca^2+^]_m_, in concert with mROS generated by the electron transport chain (ETC), triggers the opening of the low conductance mPTP and the redistribution of Ca^2+^ and H^+^ across the IMM causing a brief depolarization that temporarily shuts down mitochondrial bioenergetics and contributes to an mROS flash (Fig. 5E, steps 1–3). The reduction in [Ca^2+^]_m_ (and the entry of H^+^ into the mitochondria) favors mPTP closure, and, hence, the mPTP switches between on and off states resulting in a ΔΨ_m_ flicker^50,51^. The transient mPTP opening/ΔΨ_m_ flicker leads to an ROS burst in the MAMs^33^ (Fig. 5E, step 4), the redox-mediated IP_3_R sensitization/release of ER Ca^2+^, and the generation of the next [Ca^2+^]_m_ transient that restarts the cycle (Fig. 5E, step 4 and back to step 1).

Our study showed both the initial [Ca^2+^]_m_ rise and the subsequent transients to decrease in the presence of GsMTx4, the selective inhibitor of the cationic mechanosensitive channels Piezo1 and the transient receptor potential (TRP) channels. Piezo1 is known to mediate the SS-induced Ca^2+^ influx and the initial [Ca^2+^]_c_ rise in HUVECs^35,36^, and, since the MAMs region is continuous with the cytosol, Piezo1 is expected to, at least in part, be responsible for the initial peak of [Ca^2+^]_m_ and the subsequent [Ca^2+^]_m_ transients, in agreement with our findings. Others showed Piezo1 to interact with the sarco/endoplasmic Ca^2+^ ATPase (SERCA) and the Piezo1-mediated Ca^2+^ influx to maintain the SR Ca^2+^ content and the [Ca^2+^]_c_ sparks in cardiomyocytes^52^, suggesting that, in our case, the SS-induced Piezo1-mediated Ca^2+^ entry may help maintain the ER Ca^2+^ content and, hence, the [Ca^2+^]_m_ transients in MCU OX ECs (Fig. 5E, steps 5–6).

Our study also showed a significant inhibition of both the initial [Ca^2+^]_m_ rise and the subsequent transients by the NOS inhibitor L-NAME. Since (a) the SS-induced, Piezo1-mediated Ca^2+^ influx phosphorylates/activates eNOS^53^, (b) the produced NO, either on its own or via formation of reactive nitrogen species (RNS)/ONOO^−^, increases mROS production by the ETC^41–43^, and (c) mROS are necessary for the SS-induced [Ca^2+^]_m_ transients in MCU OX ECs (this study), it is expected that NO via mROS will enhance the formation of [Ca^2+^]_m_ transients (Fig. 5, steps 7–8). In agreement with our study, NO/RNS were found to mediate the generation of [Ca^2+^]_c_ sparks in cardiomyocytes subjected to pressurized flow^46^. However, our findings also showed L-NAME to inhibit the SS-induced initial [Ca^2+^]_m_ rise, which was unaffected by MT. Although the exact mechanism for the dependance of the initial [Ca^2+^]_m_ rise on NO/RNS is unknown, alterations in NO/RNS levels via redox-dependent posttranslational modifications were shown previously to alter the activity of plasma membrane Ca^2+^ entry channels, such as the TRP channels and store-operated Ca^2+^ channels^54^. In particular, the TRP vanilloid-type 4 (TRPV4) channel, which allows for Ca^2+^ entry in SS-exposed ECs^55^ and may be regulated by Piezo1^36^, was shown to get activated via NO-mediated cysteine S-nitrosylation^56^ and, hence, in the absence of NO, the SS-induced Ca^2+^ influx (and the [Ca^2+^]_m_ initial peak) will be compromised, in agreement with our findings.

The present study did not examine the long-term effects of SS in MCU OX ECs. However, during the 9 min of EC exposure to SS, we did not observe any global mitochondrial depolarization events (detected by TMRM) or significant mitochondrial fission events (detected by either mitoTracker Red or mito-GCaMP6). Furthermore, the [Ca^2+^]_m_ oscillations remained confined in the regions of the mitochondrial network where they originated. These observations, however, cannot exclude the possibility that, if SS were to be extended (hr-days), it might activate mitochondrial fission, inflammation, and apoptosis. MCU OX alone does not cause apoptosis, but is known to exacerbate it, when cells are exposed to various oxidant stimuli^2,57^. SS alone is known to protect ECs from apoptosis by activating nuclear factor erythroid 2-related factor (Nrf)2 and Krüppel-like factor (KLF)2^58,59^. A study found the _m_Ca^2+^ influx to be essential for agonist-induced eNOS activation^60^. If the _m_Ca^2+^ influx similarly regulates the SS-induced eNOS activation/NO production, then it is possible that exposure of MCU OX ECs to SS would generate pathological levels of NO/RNS, which could lead to apoptosis.

The mROS flash frequency is thought to correlate with the mitochondrial metabolic state, because it was shown to increase in response to increased substrate, glucose or fatty acid, supply in cells^61^, and to also increase prior to a global ROS increase, mitochondrial fission, and cytochrome c release in apoptosis^62^. Since the [Ca^2+^]_m_ transients are a part of the loop that consists of ΔΨ_m_ flickers/mROS flashes/[Ca^2+^]_m_ transients, it is reasonable to expect that the [Ca^2+^]_m_ transients regulate the exact same signaling pathways as the mROS flashes. In agreement to that, a recent study showed the [Ca^2+^]_m_ oscillations generated by laser stimulation in HeLa cells to be responsible for phosphatase and tensin homolog (PTEN)-induced kinase (PINK)1 accumulation on the outer mitochondrial membrane (OMM), parkin recruitment to the OMM, and mitophagy^63^.

The success of monitoring intracellular or intraorganellar Ca^2+^ dynamics requires that the detection range of the probe is appropriate for the size and time course of the Ca^2+^ transient(s). The GCaMP Ca^2+^ sensors were designed to probe neuronal function, because neurons have fast Ca^2+^ dynamics and relative low peak accumulations (< 1 µM)^64^. The GCaMP6 sensor employed was chosen due to its rapid kinetics and high Ca^2+^ sensitivity that contribute to an increased temporal resolution of signals. However, GCaMP6 has a relatively high Ca^2+^ affinity (K_d_ of 0.17 µM)^65,66^, which means that transient peaks close to the maximum dynamic range of ~ 1 µM will be saturated. [Ca^2+^]_m_ was previously recorded in cultured pulmonary artery ECs transfected to express the fluorescence resonance energy transfer (FRET)-based low-affinity _m_Ca^2+^ indicator Cameleon D3cpv (K_d_ of 0.76 µM) during cell exposure to histamine (100 nM)^67^. [Ca^2+^]_m_ was shown to rise from 0.1 µM at baseline to 0.8 µM immediately following histamine addition and within 1 min decreased to 0.2 µM and remained at that level at later time points^67^. Mitochondria, including EC mitochondria, have a phosphate buffering system that does not allow [Ca^2+^]_m_ to reach above 2 µM^68,69^. Based on all the above, and, since [Ca^2+^]_m_ signals were not calibrated in this study, it is possible that the relatively large [Ca^2+^]_m_ signal upon the onset of flow may be saturated and that would lead to the absence of a difference in peak amplitudes between sheared MCU-transduced ECs and sheared controls. There is no such concern regarding the mito-GCaMP6-mediated recording of the [Ca^2+^]_m_ transients following the initial flow-induced peak.

Another limitation of this study is that the [Ca^2+^]_c_ response was not monitored in MCU-transduced ECs under SS. The mito-GCaMP6 fluorescence is not compatible to double-label with Fluo-4. It is compatible with Fura-2, but Fura-2 requires a different LED light source for excitation. For the double-labeling experiments to add meaningful information on Ca^2+^ homeostasis in sheared ECs, the cytosolic and mitochondrial sensors should have similar Ca^2+^ affinities and kinetics to allow for optimal detection of large vs. small and fast vs. slow Ca^2+^ dynamics in the different compartments. Furthermore, it is possible that, in the case of the shear-induced [Ca^2+^]_m_ transients in MCU-transduced ECs, the [Ca^2+^]_c_ sensors would not detect any significant perturbations, because these small [Ca^2+^]_m_ transients would mostly affect the Ca^2+^ concentration in the MAMs.

The limited number of studies that recorded both [Ca^2+^]_c_ and [Ca^2+^]_m_ responses in the same cells found [Ca^2+^]_m_ to oscillate concomitantly with [Ca^2+^]_c_: Ishii et al. used pericam and Rhod-2 to measure [Ca^2+^]_c_ and [Ca^2+^]_m_, respectively, in HeLa cells stimulated with histamine and, based on published characteristic signals, the oscillation frequency was in the order of tens of mHz with the mitochondrial oscillations showing a time lag compared to cytosolic ones^70^. Hajnoczky et al. used Fura-2 and dihydro-Rhod-2 to measure [Ca^2+^]_c_ and [Ca^2+^]_m_, respectively, in hepatocytes stimulated with a low dose of vasopressin and, based on characteristic signals, the oscillation frequency was lower than 10 mHz^71^. Samanta et al. used Fura-2 and pericam to measure [Ca^2+^]_c_ and [Ca^2+^]_m_, respectively, in a monocytic cell line stimulated with a leukotriene and showed signals with an oscillation frequency of ~ 15 mHz^72^. In our prior work, we used Fluo-4 to study [Ca^2+^]_c_ transients/oscillations in (untransduced or Ad.βGal-transduced) HUVECs exposed to 10 dynes/cm^2^ for 5 min, and found a [Ca^2+^]_c_ oscillation frequency of ~ 17 mHz^43^, which differs from the [Ca^2+^]_m_ oscillation frequency reported in this study (~ 1 mHz) by an order of magnitude. The mismatch in [Ca^2+^]_c_ and [Ca^2+^]_m_ oscillation frequencies is not due to differences between Fluo-4 and GCaMP6, since the time course and magnitude of fluorescence response to agonists were reported to be similar for the two indicators^73^. The mismatch in oscillation frequencies is probably due to the different methods used to detect transients/oscillations: In our SS-induced [Ca^2+^]_c_ study^43^, the transients had relatively large amplitudes (mean ΔF/F_0_ of ~ 0.5) and a threshold of > 15% of the local ΔF/F_0_ was chosen arbitrarily to identify [Ca^2+^]_c_ transients. In contrast, in the present study, because of the relatively small amplitude of [Ca^2+^]_m_ transients (mean ΔF/F_0_ of < 0.1), we developed a mathematical approach to identify [Ca^2+^]_m_ transients by selecting an intensity threshold and a drop (percentage of the maximum ΔF/F_0_) and, as a result, we identified fewer events and a lower [Ca^2+^]_m_ oscillation frequency compared to the previously reported [Ca^2+^]_c_ oscillation frequency in sheared (untransduced) ECs. In sheared MCU OX ECs, our mathematical analysis yielded a [Ca^2+^]_m_ oscillation frequency of ~ 10 mHz, which is within the range of frequencies calculated from the published [Ca^2+^]_m_ characteristic signals in agonist-stimulated cells, as listed above.

In summary, this is the first study that recorded _m_Ca^2+^ signaling in human ECs with and without MCU OX during exposure to SS. Our main findings are that (a) SS leads to ΔΨ_m_ flickers/mROS flashes/[Ca^2+^]_m_ oscillations in ECs with increased MCU activity/_m_Ca^2+^ uptake and (b) the SS-induced oscillatory _m_Ca^2+^ signaling in ECs with increased MCU activity is mediated, at least in part, by Piezo1 activation, mROS and NO/RNS. Since EC MCU expression was upregulated under pathological conditions, a better understanding of the EC Ca^2+^ and redox homeostasis under conditions that mimic the in vivo hemodynamic environment may lead to development of improved prevention and/or treatment strategies for EC dysfunction and CVD.

## Materials and methods

### EC culture and adenoviral transduction

Pooled primary HUVECs (Lonza: C2519AS) were grown in complete EC growth medium (Lonza; EGM™ Endothelial Cell Growth Medium BulletKit™: CC-3124) in a tissue culture incubator at 37 °C in an atmosphere of 5% CO_2_/95% air. Plastic parallel-plate flow chamber slides with high optical quality (ibidi; μSlide 0.6 luer, 1.5 coverslip: 80186) were coated with 6% fibronectin solution for 40 min. ECs of passage 3–5 were seeded on the fibronectin-coated chamber slides at a seeding density of 36,000–40,000 cells/cm^2^. At 24 h, cells reached 60% confluency and were incubated with an adenoviral vector that expresses the mitochondria-targeted GECI mito-GCaMP6m (Ad.mito-GCaMP6; 475 nm excitation/510 nm emission) in EGM at a multiplicity of infection (MOI) of 10 for 6 h, as described previously by our group^29,30^. Ad.mito-GCaMP6 was constructed by fusing the mitochondrial matrix-targeting sequence to a DNA plasmid encoding GCaMP6m (GenBank), and then inserting the plasmid into a replication-deficient adenoviral vector under the control of the promoter-enhancer region of the human cytomegalovirus (Vector BioLabs)^29,30^. Media were changed every 24 h after the 6 h incubation period. At 48 h post-transduction, ECs had formed confluent monolayers and were either exposed to SS or kept static. Some EC cultures at 60% confluency were also transduced with Ad.MCU at an MOI of 50, as previously^31^, simultaneously with Ad.mito-GCaMP6, in EGM for 6 h with media changes every 24 h after the transduction period. Ad.MCU was made in house by inserting a human gene coding for MCU (GenBank) into a replication-deficient adenoviral vector under the control of the promoter-enhancer region of the human cytomegalovirus^31^. MCU OX was verified by Western blotting. EC extracts were prepared using RIPA buffer (Abcam: ab156034) and Halt™ protease and phosphatase inhibitor cocktail, EDTA-free (ThermoFisher Scientific: 78441). Equal amounts of protein (20 μg/lane) were separated on 4–12% Bis–Tris polyacrylamide gels (ThermoFisher Scientific: WG1402BOX), transferred to a PVDF membrane (ThermoFisher Scientific: 1B24001) using iBlot 2 PVDF regular stacks (ThermoFisher Scientific: IB21001), and probed with antibodies specific for MCU (1:3000; Cell Signaling: D2Z3B), MICU2 (1:2000; Abcam: 101465), and β-actin (1:10,000; Santa Cruz: sc-47778). ECs either not transduced with Ad.MCU or transduced with a control Ad.βGal vector at an MOI of 50 (Vector BioLabs:1080), always in the presence of Ad.mito-GCaMP6 in EGM for 6 h, were used as controls.

### EC exposure to SS and chemical treatments

EC monolayers in ibidi chamber slides at 48 h post-transduction with Ad.mito-GCaMP6, and with/without Ad.MCU or Ad.βGal, were preincubated with basal/low-serum media consisting of EBM (Lonza; EBM™ Endothelial Cell Growth Basal Medium, phenol red-free: CC-3129) supplemented with 1% FBS (Lonza: CC-4133) and antibiotics (Lonza: CC-4083) for 4 h for the cells to become quiescent and equilibrate with the perfusion media. The slides with the EC monolayers were attached to an ibidi Pump System (ibidi: 10902) using the yellow-green perfusion set (ibidi; length 50 cm, ID 6 mm, and 10 ml media reservoirs: 10964) to achieve an arterial-level SS of 12 dynes/cm^2^ (28.52 ml/min, 61.9 mbar). During SS exposure, the slides were placed in a stage-top incubator (ibidi; 37 °C, 5% CO_2_/95% air, 80% humidity: 10720) of an inverted epifluorescence microscope (Leica; DMi8: 11525569) equipped with a high speed sCMOS camera (Leica; DFC9000 GT: 11547006), an LED8 light source (Leica: 11504256), and an environmental temperature control unit (PeCon; to maintain the fluidic unit and reservoirs at 37 °C: 11533465). For the first 1 min, EC monolayers were maintained under static conditions in basal/low-serum media to establish a baseline in mito-GCaMP6 fluorescence (representative of resting [Ca^2+^]_m_). At the end of 1 min, flow (corresponding to an SS level of 12 dynes/cm^2^) started and ECs were exposed to 9 min of SS.

To delineate the role of the plasma-membrane mechanosensitive Ca^2+^ channel Piezo1 on the SS-induced _m_Ca^2+^ response, some EC monolayers, with/without MCU or βGal OX, were treated with the Piezo1 specific inhibitor Grammostola spatulata Mechanotoxin 4 (GsMTx4, D-isoform; a kind gift from Dr. T. M. Suchyna, University at Buffalo; stock solution in PBS)^38^ at a final concentration of 5 µM in basal/low-serum media for the last 5 min of the 4 h preincubation period and throughout SS exposure (1 min static followed by 9 min SS). To investigate the role of mROS on the _m_Ca^2+^ response, some EC monolayers were treated with the mitochondrial O_2_^−.^ scavenger 2-[2,2,6,6-tetramethylpiperidin-1-oxyl-4-ylamino]-2-oxoethyl triphenylphosphonium (mitoTEMPO, abbreviated MT; Sigma-Aldrich; stock solution in DMSO: SML0737) at a final concentration of either 25 nM, 100 nM, or 1 μM for the last 5 min of the 4 h preincubation and throughout SS. These concentrations were previously shown to be effective in scavenging EC mROS^39,40^. To test for the contribution of the endothelial NO synthase (eNOS) activation/NO production in the SS-induced _m_Ca^2+^ response, some EC monolayers were preincubated with the NOS inhibitor L-N^G^-nitro arginine methyl ester (L-NAME; Sigma-Aldrich; stock solution in H_2_O: N5751) at a final concentration of 500 µM for the last 30 min of the 4 h preincubation period and throughout SS, as in earlier work by our group^44^. Control ECs were treated with equal volumes of the respective vehicle, either H_2_O or DMSO (final DMSO concentration ≤ 0.01% v/v; Tocris: 3176).

### Image processing and analysis of _m_Ca^2+^ signaling

Digital images of relative fluorescence intensity changes were collected using a 63 × magnification at a frequency of 1 Hz and stored at 30 frames/s (fps) as .mpeg files using LAS X Life Science Microscope Software (Leica)*.* MATLAB (MathWorks) scripts were created to track temporal changes in mito-GCaMP6 fluorescence intensity (in arbitrary units) and were averaged over the surface area of each EC in a microscope field of view. Acquired videos were uploaded to MATLAB as a three-dimensional matrix with the first two dimensions representing the x/y dimensions of the video frame and the third one representing the acquisition time/frame number. ECs were identified and segmented using the *roipoly* function, which prompted for manual drawing of cell boundaries (from phase contrast images) and saved each cell area as a region of interest (ROI). First, an average fluorescence (grayscale) intensity was calculated for each ROI in each frame. Then, for quantification of the observed mito-GCaMP6 transients, a separate script *nnz* was used, which adopted the prior segmentation method for analysis of each ROI in each video frame. The *nnz* function counted the number of image pixels within each ROI that had an intensity level above a grayscale intensity threshold. Thresholds were selected from a 3D topographical surface of pixels above a threshold vs. a range of threshold values vs. time. As a result of this analysis, each ROI corresponded to a 600-element vector containing the pixels above the threshold at each video frame. To quantify the number of [Ca^2+^]_m_ transients from this data set, the *findpeaks* function was used. All relative maxima in the dataset were filtered using the parameter ‘*MinPeakProminence’*, which only considers relative maxima that are greater than the preceding relative minimum by a specified amount. This prominence value or, else called, drop percentage was determined by multiplying the maximum intensity value of the data set (which corresponds to the fluorescence peak right after the flow onset) by a decimal value in the range of 0.05–0.25. The exact drop value for each ROI was determined by examining the persistence of [Ca^2+^]_m_ transients over a range of drop percentages. Specifically, a value within the widest range of drop percentages with persistent [Ca^2+^]_m_ transients was selected as the drop percentage value for the data set from each ROI. This determined the number of [Ca^2+^]_m_ transients in each individual EC over the 9 min of SS exposure. The initial peak in fluorescence intensity due to the onset of SS was excluded, as it was present in the _m_Ca^2+^ response of every SS-exposed EC and was not considered a transient. The code developed for the above analysis is supplied in Supplementary Information.

Average mito-GCaMP6 fluorescence intensity for each ROI (F) was used to quantify a baseline (F_0_; time-averaged over the 1 min static prior to SS) and the peak amplitude (ΔF = F − F_0_) at each time point under SS for each of 60 ROIs (ECs from n = 4 independent experiments; each 63 × microscope image contains on average 15 ECs). Normalized fluorescence intensity (ΔF/F_0_) temporal profiles were plotted for each EC under static and SS with/without MCU OX and with/without each of the chemical treatments (GsMTx4, MT at three different concentrations, L-NAME). Average mtSOX Deep Red fluorescence intensity data per ROI were also used to plot normalized fluorescence (ΔF/F_0_) temporal profiles for each EC under SS. To accommodate for the TMRM photobleaching, data acquisition was limited to a few min, and, hence, TMRM temporal profiles were plotted as not-normalized averaged over an EC (or a subregion within a cell) fluorescence intensity data (F, in arbitrary units). The calculated number of [Ca^2+^]_m_ transients per EC from the threshold/drop analysis described in the previous section was used to determine the percentage of total cells (60 from n = 4 independent experiments) that exhibited [Ca^2+^]_m_ transients/oscillations in each condition tested. Cells with [Ca^2+^]_m_ transients were also binned based on their number of transients and expressed as a percentage of total cells. The number of transients per cell averaged over a total of 60 cells in each condition was quantified over binned 60 s time intervals. The cumulative number of [Ca^2+^]_m_ transients over the 9 min duration of SS was calculated and was used to determine the oscillation frequency (in mHz) per cell, which was then averaged over 60 cells from n = 4 independent experiments in each condition tested.

### Measurement of mROS levels and ΔΨ_m_

Some EC monolayers were treated with the mitochondrial O_2_^−.^ fluorescent indicator mtSOX Deep Red (Dojindo; 555 nm excitation/700 nm emission; stock solution in DMSO: M22425) at a final concentration of 2 µM in basal/low-serum media for the last 30 min of the 4 h preincubation period. Changes in ΔΨ_m_ were recorded during SS exposure in some EC monolayers by loading the cells with tetramethylrhodamine methyl ester (TMRM; ThermoFisher Scientific, 555 nm excitation/590 nm emission, stock solution in DMSO: M20036) that accumulates only in active mitochondria^34^, at a final concentration of 20 nM in basal/low-serum media for the last 30 min of the 4 h preincubation period. To visualize all mitochondria, some EC monolayers were treated with mitoTracker Red (ThermoFisher Scientific; 555 nm excitation/700 nm emission, stock solution in DMSO: M22425) at a final concentration of 100 nM in basal/low-serum media for the last 20 min of the 4 h preincubation period.

### Statistical analysis

Data on baseline fluorescence, peak amplitude, and the number of [Ca^2+^]_m_ transients per cell at 60-s time intervals in each treatment were presented as mean ± SE from 60 cells (these were all the cells from n = 4 independent experiments). Data on the number of cells that exhibited [Ca^2+^]_m_ transients expressed as percentage of total cells and on the oscillation frequency per cell were presented as mean ± SE from n = 4 independent experiments for each condition tested. Statistical differences were determined using a one-way analysis of variance (ANOVA) followed by a Dunnett's multiple comparisons test with a P ≤ 0.05 indicating statistical significance. All statistical analyses were performed using GraphPad Prism 9 on a PC.

## Supplementary Information

Supplementary Information 1.

Supplementary Video 1.

Supplementary Video 2.

## Data Availability

The raw datasets generated during and/or analyzed during the current study will be made available upon request to the corresponding author.

## References

[1] Baughman JM (2011). Integrative genomics identifies MCU as an essential component of the mitochondrial calcium uniporter. Nature.

[2] De Stefani D, Raffaello A, Teardo E, Szabo I, Rizzuto R (2011). A forty-kilodalton protein of the inner membrane is the mitochondrial calcium uniporter. Nature.

[3] Mallilankaraman K (2012). MICU1 is an essential gatekeeper for MCU-mediated mitochondrial Ca^2+^ uptake that regulates cell survival. Cell.

[4] Mallilankaraman K (2012). MCUR1 is an essential component of mitochondrial Ca^2+^ uptake that regulates cellular metabolism. Nat. Cell. Biol..

[5] Nemani N, Shanmughapriya S, Madesh M (2018). Molecular regulation of MCU: Implications in physiology and disease. Cell Calcium.

[6] Alevriadou BR (2021). Molecular nature and physiological role of the mitochondrial calcium uniporter channel. Am. J. Physiol. Cell Physiol..

[7] Rizzuto R, Brini M, Murgia M, Pozzan T (1993). Microdomains with high Ca^2+^ close to IP3-sensitive channels that are sensed by neighboring mitochondria. Science.

[8] Csordas G, Thomas AP, Hajnoczky G (1999). Quasi-synaptic calcium signal transmission between endoplasmic reticulum and mitochondria. EMBO J..

[9] Hajnoczky G, Csordas G, Madesh M, Pacher P (2000). The machinery of local Ca^2+^ signalling between sarco-endoplasmic reticulum and mitochondria. J. Physiol..

[10] Dong Z (2017). Mitochondrial Ca^2+^ uniporter is a mitochondrial luminal redox sensor that augments MCU channel activity. Mol. Cell.

[11] Duchen MR (1999). Contributions of mitochondria to animal physiology: from homeostatic sensor to calcium signalling and cell death. J. Physiol..

[12] Murgia M, Rizzuto R (2015). Molecular diversity and pleiotropic role of the mitochondrial calcium uniporter. Cell Calcium.

[13] Kamer KJ, Mootha VK (2015). The molecular era of the mitochondrial calcium uniporter. Nat. Rev. Mol. Cell Biol..

[14] De Stefani D, Rizzuto R, Pozzan T (2016). Enjoy the trip: Calcium in mitochondria back and forth. Annu. Rev. Biochem..

[15] Drago I, De Stefani D, Rizzuto R, Pozzan T (2012). Mitochondrial Ca^2+^ uptake contributes to buffering cytoplasmic Ca^2+^ peaks in cardiomyocytes. Proc. Natl. Acad. Sci. USA.

[16] Mammucari C (2018). Mitochondrial calcium uptake in organ physiology: From molecular mechanism to animal models. Pflugers Arch..

[17] Pathak T, Trebak M (2018). Mitochondrial Ca^2+^ signaling. Pharmacol. Ther..

[18] Scheitlin CG (2016). Endothelial mitochondria regulate the intracellular Ca^2+^ response to fluid shear stress. Am. J. Physiol. Cell Physiol..

[19] Malek AM, Alper SL, Izumo S (1999). Hemodynamic shear stress and its role in atherosclerosis. JAMA.

[20] Li YS, Haga JH, Chien S (2005). Molecular basis of the effects of shear stress on vascular endothelial cells. J. Biomech..

[21] Davies PF, Civelek M, Fang Y, Fleming I (2013). The atherosusceptible endothelium: Endothelial phenotypes in complex haemodynamic shear stress regions in vivo. Cardiovasc. Res..

[22] Simmons RD, Kumar S, Jo H (2016). The role of endothelial mechanosensitive genes in atherosclerosis and omics approaches. Arch. Biochem. Biophys..

[23] Alevriadou BR, Shanmughapriya S, Patel A, Stathopulos PB, Madesh M (2017). Mitochondrial Ca^2+^ transport in the endothelium: Regulation by ions, redox signalling and mechanical forces. J. R. Soc. Interface.

[24] Zhang Y (2021). Pyk2/MCU pathway as a new target for reversing atherosclerosis. Front. Cell. Dev. Biol..

[25] Chen LT (2021). Homocysteine induced a calcium-mediated disruption of mitochondrial function and dynamics in endothelial cells. J. Biochem. Mol. Toxicol..

[26] Chen W (2017). Importance of mitochondrial calcium uniporter in high glucose-induced endothelial cell dysfunction. Diab. Vasc. Dis. Res..

[27] Botts SR, Fish JE, Howe KL (2021). Dysfunctional vascular endothelium as a driver of atherosclerosis: Emerging insights into pathogenesis and treatment. Front. Pharmacol..

[28] Kirkman DL, Robinson AT, Rossman MJ, Seals DR, Edwards DG (2021). Mitochondrial contributions to vascular endothelial dysfunction, arterial stiffness, and cardiovascular diseases. Am. J. Physiol. Heart Circ. Physiol..

[29] Nemani N (2018). MIRO-1 determines mitochondrial shape transition upon GPCR activation and Ca^2+^ stress. Cell. Rep..

[30] Tomar D (2019). Blockade of MCU-mediated Ca^2+^ uptake perturbs lipid metabolism via PP4-dependent AMPK dephosphorylation. Cell. Rep..

[31] Tomar D (2016). MCUR1 is a scaffold factor for the MCU complex function and promotes mitochondrial bioenergetics. Cell Rep..

[32] Boyman L (2019). Dynamics of the mitochondrial permeability transition pore: Transient and permanent opening events. Arch. Biochem. Biophys..

[33] Booth DM, Varnai P, Joseph SK, Hajnoczky G (2021). Oxidative bursts of single mitochondria mediate retrograde signaling toward the ER. Mol. Cell..

[34] Elmore SP, Nishimura Y, Qian T, Herman B, Lemasters JJ (2004). Discrimination of depolarized from polarized mitochondria by confocal fluorescence resonance energy transfer. Arch. Biochem. Biophys..

[35] Wang S (2016). Endothelial cation channel PIEZO1 controls blood pressure by mediating flow-induced ATP release. J. Clin. Invest..

[36] Swain SM, Liddle RA (2021). Piezo1 acts upstream of TRPV4 to induce pathological changes in endothelial cells due to shear stress. J. Biol. Chem..

[37] Bae C, Sachs F, Gottlieb PA (2011). The mechanosensitive ion channel Piezo1 is inhibited by the peptide GsMTx4. Biochemistry.

[38] Suchyna TM (2017). Piezo channels and GsMTx4: Two milestones in our understanding of excitatory mechanosensitive channels and their role in pathology. Prog. Biophys. Mol. Biol..

[39] Dikalova AE (2010). Therapeutic targeting of mitochondrial superoxide in hypertension. Circ. Res..

[40] Dikalova AE, Kirilyuk IA, Dikalov SI (2015). Antihypertensive effect of mitochondria-targeted proxyl nitroxides. Redox Biol..

[41] Erusalimsky JD, Moncada S (2007). Nitric oxide and mitochondrial signaling: from physiology to pathophysiology. Arterioscler. Thromb. Vasc. Biol..

[42] Han Z (2009). Mitochondria-derived reactive oxygen species mediate heme oxygenase-1 expression in sheared endothelial cells. J. Pharmacol. Exp. Ther..

[43] Scheitlin CG, Nair DM, Crestanello JA, Zweier JL, Alevriadou BR (2014). Fluid mechanical forces and endothelial mitochondria: A bioengineering perspective. Cell. Mol. Bioeng..

[44] Giedt RJ, Yang C, Zweier JL, Matzavinos A, Alevriadou BR (2012). Mitochondrial fission in endothelial cells after simulated ischemia/reperfusion: Role of nitric oxide and reactive oxygen species. Free Radic. Biol. Med..

[45] Zhou L, Aon MA, Liu T, O'Rourke B (2011). Dynamic modulation of Ca^2+^ sparks by mitochondrial oscillations in isolated guinea pig cardiomyocytes under oxidative stress. J. Mol. Cell Cardiol..

[46] Kim JC, Wang J, Son MJ, Woo SH (2017). Shear stress enhances Ca^2+^ sparks through Nox2-dependent mitochondrial reactive oxygen species generation in rat ventricular myocytes. Biochim. Biophys. Acta Mol. Cell Res..

[47] Kuznetsov AV, Javadov S, Saks V, Margreiter R, Grimm M (2017). Synchronism in mitochondrial ROS flashes, membrane depolarization and calcium sparks in human carcinoma cells. Biochim. Biophys. Acta Bioenerg..

[48] Hou T (2013). Synergistic triggering of superoxide flashes by mitochondrial Ca^2+^ uniport and basal reactive oxygen species elevation. J. Biol. Chem..

[49] Yeh LH (1999). Shear-induced tyrosine phosphorylation in endothelial cells requires Rac1-dependent production of ROS. Am. J. Physiol..

[50] Feng G, Liu B, Hou T, Wang X, Cheng H (2017). Mitochondrial flashes: Elemental signaling events in eukaryotic cells. Handb. Exp. Pharmacol..

[51] Bonora M, Giorgi C, Pinton P (2022). Molecular mechanisms and consequences of mitochondrial permeability transition. Nat. Rev. Mol. Cell. Biol..

[52] Jiang F (2021). The mechanosensitive Piezo1 channel mediates heart mechano-chemo transduction. Nat. Commun..

[53] Jin YJ (2021). Protein kinase N2 mediates flow-induced endothelial NOS activation and vascular tone regulation. J. Clin. Invest..

[54] Trebak M, Ginnan R, Singer HA, Jourd'heuil D (2010). Interplay between calcium and reactive oxygen/nitrogen species: An essential paradigm for vascular smooth muscle signaling. Antioxid. Redox Signal.

[55] Lu T (2017). Role of the endothelial caveolae microdomain in shear stress-mediated coronary vasorelaxation. J. Biol. Chem..

[56] Yoshida T (2006). Nitric oxide activates TRP channels by cysteine S-nitrosylation. Nat. Chem. Biol..

[57] Liao Y (2015). Mitochondrial calcium uniporter protein MCU is involved in oxidative stress-induced cell death. Protein Cell.

[58] Dai G (2007). Biomechanical forces in atherosclerosis-resistant vascular regions regulate endothelial redox balance via phosphoinositol 3-kinase/Akt-dependent activation of Nrf2. Circ. Res..

[59] Wang N (2006). Shear stress regulation of Kruppel-like factor 2 expression is flow pattern-specific. Biochem. Biophys. Res Commun..

[60] Charoensin S (2017). Intact mitochondrial Ca^2+^ uniport is essential for agonist-induced activation of endothelial nitric oxide synthase (eNOS). Free Radic. Biol. Med..

[61] Gong G, Liu X, Zhang H, Sheu SS, Wang W (2015). Mitochondrial flash as a novel biomarker of mitochondrial respiration in the heart. Am. J. Physiol. Heart Circ. Physiol..

[62] Ma Q (2011). Superoxide flashes: Early mitochondrial signals for oxidative stress-induced apoptosis. J. Biol. Chem..

[63] Yu Z (2021). Mitochondrial Ca(2+) oscillation induces mitophagy initiation through the PINK1-Parkin pathway. Cell Death Dis..

[64] Chen TW (2013). Ultrasensitive fluorescent proteins for imaging neuronal activity. Nature.

[65] Ohkura M (2012). Genetically encoded green fluorescent Ca^2+^ indicators with improved detectability for neuronal Ca2+ signals. PLoS ONE.

[66] Suzuki J, Kanemaru K, Iino M (2016). Genetically encoded fluorescent indicators for organellar calcium imaging. Biophys. J..

[67] Marcu R, Wiczer BM, Neeley CK, Hawkins BJ (2014). Mitochondrial matrix Ca^2+^ accumulation regulates cytosolic NAD^+^/NADH metabolism, protein acetylation, and sirtuin expression. Mol. Cell Biol..

[68] Graier WF, Frieden M, Malli R (2007). Mitochondria and Ca^2+^ signaling: Old guests, new functions. Pflugers Arch..

[69] Finkel T (2015). The ins and outs of mitochondrial calcium. Circ. Res..

[70] Ishii K, Hirose K, Iino M (2006). Ca^2+^ shuttling between endoplasmic reticulum and mitochondria underlying Ca^2+^ oscillations. EMBO Rep..

[71] Hajnoczky G, Robb-Gaspers LD, Seitz MB, Thomas AP (1995). Decoding of cytosolic calcium oscillations in the mitochondria. Cell.

[72] Samanta K, Douglas S, Parekh AB (2014). Mitochondrial calcium uniporter MCU supports cytoplasmic Ca^2+^ oscillations, store-operated Ca^2+^ entry and Ca^2+^-dependent gene expression in response to receptor stimulation. PLoS ONE.

[73] Wu N, Nishioka WK, Derecki NC, Maher MP (2019). High-throughput-compatible assays using a genetically-encoded calcium indicator. Sci. Rep..

