# Supplementary material for: The mitochondrial Ca2+ uniporter channel synergizes with fluid shear stress to induce mitochondrial Ca2+ oscillations
Source: Sci Rep. 2022 Dec 7;12:21161. doi: 10.1038/s41598-022-25583-7 (PMC9729216; doi:10.1038/s41598-022-25583-7)
Supplement: Supplementary file 1 — Supplementary Information 1. [file 41598_2022_25583_MOESM1_ESM.pdf]

**Supplementary Information**

"The Mitochondrial $Ca^{2+}$ Uniporter channel synergizes with fluid shear stress to induce mitochondrial $Ca^{2+}$ oscillations" by Patel A et al.

**Supplementary Figure 1** (related to Figure 1D). Original/uncropped blots of lysates from untransduced, Ad.βGal- or Ad.MCU-transduced (all static) ECs against MCU, MICU2, and β-actin (15 μg total protein per lane).

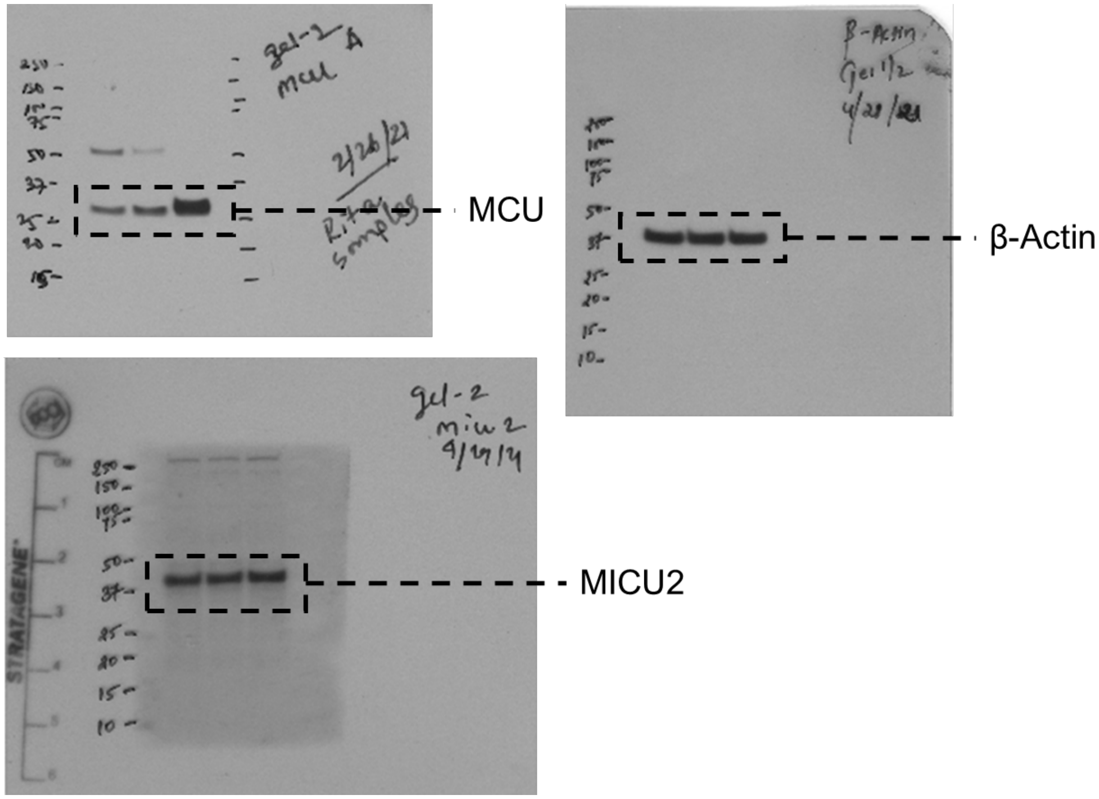

**Supplementary Video 1** (related to Figure 1C). Video (titled SS.mp4) of mito-GCaMP6 fluorescence response in (untransduced) ECs exposed to 1 min of static incubation followed by 9 min of SS. The video was captured at 1 frame/s and was saved as MP4 (30 frames/s). The supplementary video 1 is provided as a separate file.

**Supplementary Video 2** (related to Figure 1E). Video (titled AdMCU-SS.mp4) of mito-GCaMP6 fluorescence response in Ad.MCU-transduced ECs exposed to 1 min of static incubation followed by 9 min of SS. The video was captured at 1 frame/s and was saved as MP4 (30 frames/s). The supplementary video 2 is provided as a separate file.

**Supplementary Code** (related to Figures 2 and 5). A MATLAB script CodeS1_AnalysisofTransients.m was developed to import videos, analyze them for average fluorescence intensity, and quantify the number of transients per cell over time along with the cumulative number of transients per cell. The script also includes functions to generate pseudo-3D fluorescent images, 3D topography surfaces of $\log_{10}$(number of pixels) vs. threshold intensity vs. time, and plot pixels above threshold vs. time, drop persistence, and cumulative distribution functions. The script in pdf is included below.

**Average_Intensity.m**

```
%%
%the video file and ROI file must share the same prefix.
%If ROIs are only generated by this code or Flashing_Pixel_Count, this
will
%happen automatically
clc
clear
%% Select video to be analyzed
% must be in current folder

input_video_fname = '12min_video.mp4'; % set default filename

%prompt to select input video file (uses default name)
[input_video_fname,input_video_path] = uigetfile('*.mp4',...
    'Select the input video file',input_video_fname);
if isequal(input_video_fname,0) || isequal(input_video_path,0)
   disp('User selected Cancel')
   return;
else
   fprintf('Input Video: "%s%s"
\n',input_video_path(end),input_video_fname);
end
% can be video directly from microscope, or post-processed with
% binarization, etc.

filename = erase(input_video_fname,'.mp4');

%Try and keep prefixes consistent across file formats
%ex. video files, ROIs, histogram data

roi_filename = strcat(filename,'_ROI.mat');

roi_exists = input('Does a properly named ROI exist already? Type 0
for no, 1 for yes: ');
% loads ROIs if they were drawn previously. If not, prompts their
creation.
if roi_exists == 1

    load(roi_filename)
    z = length(ROI_array)+1;
    % Load the input mp4 file and save as a 3D matrix:
    obj = VideoReader(input_video_fname);
    vid = read(obj);
else
    % Load the input mp4 file and save as a 3D matrix:
    obj = VideoReader(input_video_fname);
    vid = read(obj);
    figure(1)
    template = vid(:,:,:,100); %selects frame with bright/focused
signal
```

```
    %4th index (frame #) can be changed if clarity is better in
another
    %video frame
    phase = rgb2gray(template);
    figure(1)
    imshow(phase) % display video frame for drawing
    ROI_array = {}; % initializes empty array

    % Selection instructions:
    % cursor appears on image
    % Click to add polygon vertices, lines appear automatically
    % Once a closed polygon is created by clicking on the first
vertex,
    % double click to save region of interest. Repeat for all cells
fully
    % inside frame

    z = 1;
    status = 1;
    while status == 1
        % counter
        z

        new_ROI = roipoly;      % draw polygon around cell
        ROI_array{z} = new_ROI; % save ROI to cell array for later
        %base_ROI = base_ROI + new_ROI; %combines all into one image
        phase = phase.*uint8(imcomplement(new_ROI));
        imshow(phase)
        z = z + 1;
        status = input('Type 1 to Continue, 0 to Stop: ');
    end

    roi_filename = strcat(filename,'_ROI.mat');
    save(roi_filename,'ROI_array') % saves ROIs for future use
end
%% Loop
tic
% Preallocate final output array
Intensity_Regions = zeros(z-1,600);
Intensity_Regions_Norm = zeros(z-1,600);
% Loop through the frames:
for i=1:600

    % Print the counter:
    i

    % Load each frame as a grayscale image I
    I = rgb2gray(vid(:, :, :, i));


    for j = 1:z-1
```

```
        % counter
        % j

        current_ROI = uint8(I).*uint8(ROI_array{j});
        Intensity_Regions(j,i) = 
sum(sum(current_ROI))/nnz(current_ROI);


    end
    toc
end


%% export to Excel
output_excel_fname = strcat(filename,'.xlsx'); %can add additional 
modifiers to excel filename if necessary
for k = 1:600
    Intensity_Regions_Norm(:,k) = (Intensity_Regions(:,k)-
Intensity_Regions(:,1))./Intensity_Regions(:,1);
end
A = vertcat(1:1:600,Intensity_Regions,1:1:600,Intensity_Regions_Norm);
writematrix(A,output_excel_fname,'Sheet',1,'Range','A1')
```

**Flashing_Pixel_Count.m**

```
%%
%the video file and ROI file must share the same prefix.
%If ROIs are only generated by this code or Average_Intensity, this 
will
%happen automatically
clc
clear
%% Select video to be analyzed
% must be in current folder

input_video_fname = '12min_video.mp4'; % set default filename

%prompt to select input video file (uses default name)
[input_video_fname,input_video_path] = uigetfile('*.mp4',...
    'Select the input video file',input_video_fname);
if isequal(input_video_fname,0) || isequal(input_video_path,0)
   disp('User selected Cancel')
   return;
else
   fprintf('Input Video: "%s%s"
\n',input_video_path(end),input_video_fname);
end
% can be video directly from microscope, or post-processed with
% binarization, etc.
```

```
filename = erase(input_video_fname,'.mp4');

%Try and keep prefixes consistent across file formats
%ex. video files, ROIs, histogram data

roi_filename = strcat(filename,'_ROI.mat');
threshold = 100*zeros(1,25); % sets universal intensity threshold.
                            % can adjust specific cells later
adj_cells = input('Enter #s of cells w/ adjusted thresholds, as
vector, type 0 if none: ');
if adj_cells ~= 0
    for a = adj_cells
        thresholds = input('Enter new threshold for cell: ');
        threshold(a) = thresholds;
        % sets a new threshold for specific cells
    end
  end

roi_exists = input('Does a properly named ROI exist already? Type 0
for no, 1 for yes: ');
% loads ROIs if they were drawn previously. If not, prompts their
creation.
if roi_exists == 1

    load(roi_filename)
    z = length(ROI_array)+1;
    % Load the input mp4 file and save as a 3D matrix:
    obj = VideoReader(input_video_fname);
    vid = read(obj);
else
    % Load the input mp4 file and save as a 3D matrix:
    obj = VideoReader(input_video_fname);
    vid = read(obj);
    figure(1)
    template = vid(:,:,:,100); %selects frame with bright/focused
signal
    %4th index (frame #) can be changed if clarity is better in
another
    %video frame
    phase = rgb2gray(template);
    figure(1)
    imshow(phase) % display video frame for drawing
    ROI_array = {}; % initializes empty array

    % Selection instructions:
    % cursor appears on image
    % Click to add polygon vertices, lines appear automatically
    % Once a closed polygon is created by clicking on the first
vertex,
    % double click to save region of interest. Repeat for all cells
fully
    % inside frame
```

```
    z = 1;
    status = 1;
    while status == 1
        % counter
        z

        new_ROI = roipoly;       % draw polygon around cell
        ROI_array{z} = new_ROI; % save ROI to cell array for later
        %base_ROI = base_ROI + new_ROI; %combines all into one image
        phase = phase.*uint8(imcomplement(new_ROI));
        imshow(phase)
        z = z + 1;
        status = input('Type 1 to Continue, 0 to Stop: ');
    end

    roi_filename = strcat(filename,'_ROI.mat');
    save(roi_filename,'ROI_array') % saves ROIs for future use
end

%% Acquire raw data- pixels above thresholds

all_npx = zeros((z-1),600);             %preallocates for all
collected data
                                        %number of cells x number of
frames
max_frame = rgb2gray(vid(:,:,:,75));    %selects frame w/ approximate
peak intensities
cell_max_intensity = zeros(1,z-1);      %preallocates for ROI data
cell_area = zeros(1,z-1);

for j = 1:z-1                           %loop through cells
    stats = regionprops(ROI_array{j},max_frame,'MaxIntensity','Area');
    %retrieves ROI data for later
    cell_max_intensity(j) = stats(1).MaxIntensity;
    cell_area(j) = stats(1).Area;
    %saves data to array
end

for i = 1:600  %loop through frames

    i %counter (good estimate of code speed)

    I = rgb2gray(vid(:, :, :, i));  %load grayscale image

    for j = 1:z-1   %loop through cells

        %a = cell_max_intensity(j);  %load cell's max intensity
        I2 = I.*uint8(ROI_array{j}); %isolate single cell

        all_npx(j,i) = nnz(I2>threshold(j)); %count pixels above
threshold
```

```
    end
end
%% Create CDF
drp = 0.10; %this number can be set anywhere from 0.10 to 0.25.

%If different cells require different drops, assign an array to 'drp'
%instead of a single value, and in the below loop call 'drp(j)'
instead of
%'drp'

                              %define peak by percent drop relative to
max value
pks_and_locs = {};            %cell array for each cell's peaks and
peak locations

for j = 1:z-1
    npx1 = all_npx(j,:); %loads pixel count for half max threshold
    [pks1,locs1] = findpeaks(npx1,'MinPeakProminence',max(npx1).*drp);
    %finds peak values and locations based on chosen drop percentage
    pks_and_locs{1,j} = pks1;
    pks_and_locs{2,j} = locs1;
    %save values to cell array
end

st = zeros(z-1,600);                       %preallocate cell array for
distribution data
for j = 1:(z-1) %loop through cells
    npx1 = all_npx(j,:);             %loads pixel count for threshold
    st1 = zeros(1,size(npx1,2));     %preallocates distribution for
cell
    locs1 = pks_and_locs{2,j};       %loads peak locations for cell
    for i = 1:1:size(locs1,2)        %loop through # of peaks
        st1(locs1(i):end)=st1(locs1(i):end) +
ones(1,size([locs1(i):size(npx1,2)],2));
        %builds graph by adding height at the time of each peak
    end
    if max(npx1./cell_area(j)) < 0.0025
        st1 = zeros(1,size(npx1,2));
    end
    st(j,:) = st1; %saves single plot to cell array
end

histo_filename = strcat(filename,'_st_final.mat');
save(histo_filename,'st')

%here is the current final output of the code (MAT-file). Prefixes and
file format can
%be adjusted.
```

**Threshold_Plots_3D.m**

```
clc
clear
filename = input('Enter Filename: ','s');

vid_name = strcat(filename,'.mp4');

obj = VideoReader(vid_name);
vid = read(obj);
load(strcat(filename,'_ROI.mat'))

%%
%cell_num = input('Enter Cell Number: ');
%cell_num = [7];
for k = 1:1

    % Load the masks for the ROIs:
    %load('ROIs4.mat')

    % Initialize time series
    npxs = zeros(600,180);

    tic
     for i=1:1:600

        I = rgb2gray(vid(:, :, :, i));
        %Cells: 1,5,7,12,14
        I4 = I.*uint8(ROI_array{2});

        for j=0:1:179

            % Print the counters:
            ['(i,j) = (',num2str(i),',',num2str(j),')']

            npxs(i,j+1) = nnz(I4>j);

        end
     end
    toc


    figure('DefaultAxesFontSize',18)
    % f = figure;
    % u = f.WindowState;
    %f.WindowState = 'maximized';
    [x,y] = meshgrid([0:1:179],[0:1:599]);
    surf(x,y,log10(npxs))
    lighting phong
    shading interp
    colormap turbo
    set(gcf,'color','w');
```

```
    grid on
    view(-34,80)
    xlabel('Threshold')
    xticks(0:50:180)
    ylabel('Time')
    yticks(0:60:600)
    %title('Vid 6 Cell 14')

    figure(k)
    print(gcf,'log_pixels_3D.jpg','-djpeg','-r300');
    ax = gca;

    fig_name = strcat(filename,'_',num2str(2),'.jpg');
    exportgraphics(ax,fig_name,'Resolution',600)
end



%% Histogram 1
%load('admcu_sum.mat')
%all_admcu_flashes = sum(admcu_flashes);
a = gsmt_sum;
step = 60;

st_pt1 = a(1:step:end);
st_pt1 = diff(st_pt1);
st_pt1 = [st_pt1 a(end)-a(end-step+1)];

st_pt = st_pt1'/60;

figure(1)
set(gcf,'color','w');
bar(st_pt)
grid on
xticklabels({'1-60','61-120','121-180','181-240','241-300','301-
360','361-420','421-480','481-540','541-600'})
xlabel('Time')
ylabel('Flash Events/Cell')

%% histo 2
figure(2)
b = admcu_stack;
set(gcf,'color','w');
grid on

hg = [];

num_of_stvs = 60;
for i = 1:1:num_of_stvs
    if size(hg,2) < b(i,end)
        hg(b(i,end)) = 1;
    else
```

```
        hg(b(i,end)) = hg(b(i,end)) + 1;
    end
end
% step = 3;
% hg2 = hg(1:step:end);


bar(hg,'FaceColor',[0,0,0.4])
grid on
%%

tally1 = a(:,end);
tally2 = b(:,end);
tally3 = c(:,end);
tally4 = d(:,end);
tally5 = e(:,end);
tally6 = f(:,end);
tally7 = g(:,end);
tally8 = h(:,end);


%%
tally =
cat(1,tally1,tally2,[tally3,zeros(1,8)],tally4,tally5,tally6,[tally7,z
eros(1,2)],[tally8,zeros(1,8)])'./540;
%%

%%

h1 = histogram(tally1);
h1.BinWidth = 3;
a1 = h1.BinCounts;
h2 = histogram(tally2);
h2.BinWidth = 3;
a2 = h2.BinCounts;
h3 = histogram(tally3);
h3.BinWidth = 3;
a3 = h3.BinCounts;
h4 = histogram(tally4);
h4.BinWidth = 3;
a4 = h4.BinCounts;
h5 = histogram(tally5);
h5.BinWidth = 3;
a5 = h5.BinCounts;
h6 = histogram(tally6);
h6.BinWidth = 3;
a6 = h6.BinCounts;
h7 = histogram(tally7);
h7.BinWidth = 3;
a7 = h7.BinCounts;
h8 = histogram(tally8);
h8.BinWidth = 3;
```

```
a8 = h8.BinCounts;
%%
figure(4)
bar([a1;a2;a3;a4;a5;a6;a7;a8]')
xlabel('# of flash events')
%xticklabels(['0-2','3-5','6-8','9-11','12-14','15-17','18-20','21-
23','24-26'])
ylabel('# of cells')
legend('AdMCU','CsA','GsMTx','LNAME','MT 1um','MT 25 nm','MT 100
nm','Control')



%% Histogram 2

% 2nd histogram

a2 = sum(a);
b1 = sum(b);
c1 = sum(c);
d1 = sum(d);
e1 = sum(e);
f1 = sum(f);
g1 = sum(g);
h1 = sum(h);
step = 60;

st_pt1 = a2(1:step:end);
st_pt1 = diff(st_pt1);
st_pt1 = [st_pt1 a2(end)-a2(end-step+1)];

st_pt2 = b1(1:step:end);
st_pt2 = diff(st_pt2);
st_pt2 = [st_pt2 b1(end)-b1(end-step+1)];

st_pt3 = c1(1:step:end);
st_pt3 = diff(st_pt3);
st_pt3 = [st_pt3 c1(end)-c1(end-step+1)];

st_pt4 = d1(1:step:end);
st_pt4 = diff(st_pt4);
st_pt4 = [st_pt4 d1(end)-d1(end-step+1)];

st_pt5 = e1(1:step:end);
st_pt5 = diff(st_pt5);
st_pt5 = [st_pt5 e1(end)-e1(end-step+1)];

st_pt6 = f1(1:step:end);
st_pt6 = diff(st_pt6);
st_pt6 = [st_pt6 f1(end)-f1(end-step+1)];

st_pt7 = g1(1:step:end);
```

```
st_pt7 = diff(st_pt7);
st_pt7 = [st_pt7 g1(end)-g1(end-step+1)];

st_pt8 = h1(1:step:end);
st_pt8 = diff(st_pt8);
st_pt8 = [st_pt8 h1(end)-h1(end-step+1)];



% MATLAB specific
%%
st_pt = [st_pt1/60; st_pt2/60; st_pt3/53; st_pt4/60; st_pt5/60;
st_pt6/60; st_pt7/60; st_pt8/60];
st_pt = st_pt';

figure(5)
set(gcf,'color','w');
bar(st_pt)
grid on
legend('AdMCU','CsA','GsMTx','LNAME','MT 1um','MT 25 nm','MT 100
nm','Control')
xticklabels({'1-60','61-120','121-180','181-240','241-300','301-
360','361-420','421-480','481-540','541-600'})
xlabel('Time')
ylabel('Flash Events/Cell')
title('Flash Frequency')



%% Persistence Plots
%requires predefined 'thresholds' variable
for l = 1:length(videos)
    filename = videos{l};

    vid_name = strcat(filename,'.mp4');
    clear obj
    clear vid
    obj = VideoReader(vid_name);
    vid = read(obj);
    load(strcat(filename,'_ROI.mat'))
    thresh = thresholds{l};
    st_all = {};
    for k = 1:length(ROI_array)

        npx4 = [];

        for i=1:1:size(vid,4)

            i
            I = rgb2gray(vid(:, :, :, i));
            I4 = I.*uint8(ROI_array{k});
            npx4 = [npx4 nnz(I4>thresh(k))];
```

```
        end

        ndrps = 35;
        st = zeros(size(vid,4),ndrps);

        for j = 1:1:ndrps

            drp = 0.01*j;
            [pks4,locs4] =
findpeaks(npx4,'MinPeakProminence',max(npx4).*drp);
            st4 = zeros(1,size(npx4,2));
                for i=1:1:size(locs4,2)
                    ['(i,j) = (',num2str(i),',',num2str(j),')']
                    st4(locs4(i):end)=st4(locs4(i):end) +
ones(1,size([locs4(i):size(npx4,2)],2));
                end

                st(:,j) = st4;

        end
        st_all{k} = st;



    end
    st_all_all{l} = st_all;
    %% Create subplot
    figure('Position',[25 25 1500 750])
    for i = 1:length(ROI_array)
        st = st_all{i};
        subplot(3,7,i)
        set(gcf,'color','w');
        for j=1:1:10
            plot(0.01.*[1:1:35],st(j*60,:),'LineWidth',1.5)
            hold on
        end
        title(num2str(i))
        grid on
        hold off
    %     if i == 1
    %         lh =
legend('t=60','t=120','t=180','t=240','t=300','t=360','t=420','t=480',
't=540','t=600');
    %         lh.Position = [1300 600 100 100];
    %     end
    end

    sgtitle(filename)
    ax = gcf;
    fig_name = strcat(filename,'_pers','.jpg');
    exportgraphics(ax,fig_name,'Resolution',600)
end
```

```
%%
save('all_peaks.mat','st_all_all','-append')
```
